# Genetic investigation of childhood vascular tumor biology reveals pathways for therapeutic intervention

**DOI:** 10.12688/f1000research.16160.1

**Published:** 2019-04-30

**Authors:** Shayan Cheraghlou, Young Lim, Keith Choate

**Affiliations:** 1 Department of Dermatology, Yale School of Medicine, New Haven, CT, USA; 2 Department of Pathology, Yale School of Medicine, New Haven, CT, USA; 3 Department of Genetics, Yale School of Medicine, New Haven, CT, USA

**Keywords:** vascular tumors; vascular tumor management; genetics; hemangioma; Ras; MAPK; GNA14; GNA11; GNAQ; IDH

## Abstract

Vascular tumors are neoplasms of endothelial cells, a significant number of which present in childhood. Recent studies have examined the mutational landscape of many subtypes of vascular tumors, identifying mutations primarily within the Ras–mitogen-activated protein kinase (MAPK) pathway and providing a unique opportunity to consider targeted therapeutics. This review will summarize the current understanding of childhood vascular tumor pathobiology.

## Introduction

Vascular anomalies are divided into two groups: malformations and tumors ^1^. The most recent International Society for the Study of Vascular Anomalies (ISSVA) classification for vascular tumors further subdivides them into three primary groups: benign tumors, locally aggressive or borderline tumors, and malignant tumors ( Table 1).

**Table 1.** International Society for the Study of Vascular Anomalies (ISSVA) classification of vascular tumors ^23^.

Benign vascular tumors	Infantile hemangiomas
	Congenital hemangioma (CH)
	Rapidly involuting CH
	Non-involuting CH
	Partially involuting CH
	Tufted angioma
	Spindle cell hemangioma
	Epithelioid hemangioma
	Lobular capillary hemangiomas (pyogenic granuloma)
	Others
**Locally aggressive or borderline vascular tumors**	Kaposiform hemangioendothelioma
	Retiform hemangioendothelioma
	Papillary intralymphatic angioendothelioma
	Composite hemangioendothelioma
	Pseudomyogenic hemangioendothelioma
	Polymorphous hemangioendothelioma
	Hemangioendothelioma and not otherwise specified
	Kaposi sarcoma
	Others
**Malignant vascular tumors**	Angiosarcoma
	Epithelioid hemangioendothelioma
	Others

## Infantile hemangiomas

Infantile hemangioma (IH) is the most common childhood vascular neoplasm; IH has an incidence of approximately 4.5% by 3 months of age ^2^. However, the genetic mechanism of IH pathobiology remains unknown despite its prevalence. In 1999, Walter *et al*. mapped a familial form of IH to 5q31-33, housing three candidate genes—fibroblast growth factor receptor-4 ( *FGFR4*), platelet-derived growth factor receptor-β ( *PDGF-β*), and fms-related tyrosine kinase-4 ( *FLT4*) ^3^—and in subsequent work found that a small number of IHs (2 out of 15 studied cases) harbor somatic mutations in vascular endothelial growth factor ( *VEGF*)-receptor 2 (p.P1147S) and *VEGFR3* (p.P954S) (also known as *FLT4*) ^4^. Nonetheless, these variants have yet to be confirmed via *in vitro* or *in vivo* studies to cause vascular tumors or oncogenic transformation. However, a recent single-nucleotide polymorphism (SNP) study of *VEGFR-2* and *VEGF-A* in IH ** was unable to detect variants associated with disease, although the G allele of rs2010963 in *VEGF-A* was associated with a significantly lower risk of IH ^5^. Additionally, there is some debate regarding the sporadic or familial etiology of IH. Whereas twin studies suggest extra-genetic factors as the primary cause of disease, recent work studying multiple pedigrees suggests an either autosomal dominant or maternally transmitted inheritance pattern ^6, 7^. Genetic analysis of syndromic forms of IH, including PHACE syndrome (posterior fossa malformations, infantile hemangiomas, arterial anomalies, cardiac defects, and eye anomalies syndrome), which appears more often in female offspring, suggests a possible X-linked recessive pattern but has not identified a somatic mutation associated with most cases ^8– 10^. Similarly, a causative mutation has not yet been identified in LUMBAR syndrome (lower body hemangioma, urogenital malformation, myelopathy, bony deformities, anorectal malformations, arterial anomalies, and renal anomalies syndrome ^11^. The three leading hypotheses on the pathogenesis of IH are (1) local hypoxemia leading to hypoxia-inducible factor 1 alpha (HIF-1α)-induced proliferation ^12, 13^, (2) embolization of placental cells ^14– 17^, and (3) vasculogenesis/angiogenesis driven by hypoxemia-induced differentiation of mesenchymal stem cells into endothelial cells and Notch-mediated differentiation of mesenchymal stem cells into proangiogenic pericytes ^18– 22^. These hypotheses do not address the multi-system defects found in PHACE or LUMBAR syndromes which suggest that somatic mosaicism plays a role in pathogenesis.

Unlike other childhood vascular tumors, IH responds to beta-blockers; 60% of patients experience complete or near-complete resolution of the lesion and 88% of patients demonstrate improvement following a 6-month course of propanolol at a dose of 3 mg/kg per day ^24^. Prior to the advent of beta-blockers in the treatment of IH, treatment with systemic corticosteroids was considered the standard of care, and a pooled meta-analysis estimated that 69% of lesions respond to therapy ^25^, although significant morbidity—including Cushingoid features, gastroesophageal reflux, hypertension, ulceration, bleeding, failure to thrive, hirsutism, hypercholesterolemia, and infection—was also reported ^26, 27^. IH can also be distinguished from other tumors by its positive GLUT1 immunoreactivity; up to 97% of lesions show positive signal ^17, 28^. Notably, however, about half of the vessels in a given tumor are GLUT1-negative, suggesting that a heterogeneous population of endothelial cells populates these lesions, an idea later confirmed by *in vitro* studies of cells isolated from IH samples ^17, 29^. Although the genetic factors contributing to IH pathobiology remain unknown, many somatic mutations associated with GLUT1-negative vascular tumors have been identified in recent years, most in genes already known to be implicated in tumorigenesis.

## IDH1/IDH2

Genetic insight into spindle cell hemangiomas (SCHs) came from studies of Maffucci syndrome (Spranger type II enchondromatosis), a subtype of enchondromatosis presenting with multiple SCHs in early childhood ^30^. In an analysis of 13 patients with Maffucci syndrome, Pansuriya *et al*. found that 70% of SCHs had p.R132C mutations in exon 4 of isocitrate dehydrogenase 1 ( *IDH1*) ^31^. Given the disorder’s unilateral distribution of the endochondromas, the lack of mutations detected in adjacent non-lesional tissue, the absence of transmission within pedigrees, and the identification of tissue-specific *IDH1* mutations, somatic mosaicism was considered causal. Further work in sporadic, acquired SCH found that *IDH1* p.R132C is found in at least 64% of cases ^32^. Among cases negative for *IDH1* p.R132C, 20% had mutations at arginine 172 in exon 4 of *IDH2*, suggesting genetic heterogeneity ^32^.

Mutations in exon 4 at arginine 132 of *IDH1* or at arginine 140 or 172 of *IDH2* lead to the production of 2-hydroxyglutarate, an oncometabolite which causes a hypermethylation phenotype leading to the inhibition of genes responsible for terminal differentiation ^33– 36^. Additionally, mutations in *IDH1* found in gliomas lead to reduction of alpha-ketoglutarate production, inducing HIF-1α, which drives tumor growth via the hypoxia pathway ^37^. Interestingly, analysis of HIF-1α in SCH revealed a lack of expression in all samples ^32^, suggesting that *IDH1* and *IDH2* mutations driving SCH may act via a distinct mechanism.

## CAMTA1/TFE

Of childhood vascular tumors, epithelioid hemangioendothelioma (EHE) is the most common malignant variety. In 2001, Mendlick *et al*. reported an identical chromosomal translocation of t(1;3) (p36.3;q25) in two cases of EHE ^38^. Owing to low tumor cellularity and the absence of EHE cell lines, the specific genes disrupted via the translocation remained unknown until 2011, when Tanas *et al*. employed RNA sequencing to identify a fusion between the promotor region of WW domain-containing transcription regulator 1 ( *WWTR1*) on 3q25 and the carboxyl terminus of calmodulin-binding transcription activator 1 ( *CAMTA1*) on 1p36 ^39^. Given the high activity of the *WWTR1* promoter in endothelial cells and the ectopic expression of *CAMTA1*, which is typically found only in brain tissue, the authors hypothesized that *WWTR1/CAMTA1* functions as an oncogene via a promoter switch mechanism. Further work found that the *WWTR1-CAMTA1* fusion is a consistent genetic finding in EHEs of different anatomic subsites ^40^.

In EHE samples without a *WWTR1-CAMTA1* mutation, a distinct gene fusion between transcription factor E3 ( *TFE3*) and yes-associated protein 1 ( *YAP1*) was identified ^41^. Given the structural and functional similarities between *YAP1* and *WWTR1* as well as the oncogenic nature of TFE3 with preserved transcriptional activation domains, well recognized in other cancers ^42– 44^, a promoter switch similar to that of *WWTR1-CAMTA1* fusions is hypothesized to underlie oncogenesis in cases with *YAP1-TFE3* fusions.

## GNA family

**Figure 1.**
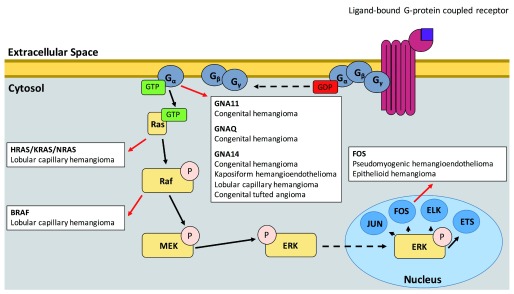
Positions of the Ras-MAPK pathway wherein mutations have been demonstrated to lead to childhood vascular tumors (red arrows). Unbroken arrows indicate activation while broken arrows indicate migration.

In recent years, a number of studies have highlighted the importance of the Ras–mitogen-activated protein kinase (MAPK) pathway in the oncogenic transformation of many childhood vascular tumors ( Figure 1). The most upstream portion of the pathway elucidated thus far is the guanine nucleotide-binding protein subunit alpha q (Gαq) family of genes: *GNAQ*, *GNA11*, and *GNA14*. In 2016, three studies demonstrated that somatic activating mutations in these genes are found in congenital hemangioma (including both rapidly involuting congenital hemangiomas and non-involuting congenital hemangiomas), kaposiform hemangioendotheliomas (KHEs), congenital tufted angiomas (TAs), and childhood lobular capillary hemangiomas (LCHs) via whole-exome sequencing ^45– 47^. Activating mutations at the arginine 183 position of *GNA11* and the glutamine 209 position of *GNA11* and *GNAQ* were also found in several cases of sporadic congenital hemangioma, while mutation of glutamine 205 in *GNA14*, the analogous position of glutamine 209 in *GNA11* and *GNAQ*, was found in one case each of KHE, LCH, and TA ^45, 46^. Recent work has also identified mutations in the glutamine 205 position of *GNA14* and the glutamine 209 position of *GNAQ* in anastomosing hemangiomas ^48^. Although these mutations have been demonstrated to cause cell morphology changes, upregulation of biochemical growth pathways, and acquisition of growth factor independence *in vitro*, they have not yet been shown to cause disease in an *in vivo* model. Notably, arginine 183 mutation in *GNAQ* is thought to be responsible for up to 88% of Sturge–Weber syndrome which presents with port-wine stains (PWSs) and leptomeningeal vascular malformations, while similar *GNA11* and *GNAQ* activating mutations underlie 77% of capillary malformations, 46% of uveal melanomas, and 83% of blue nevi ^49– 52^. Activated GNA11 and GNAQ are thought to mediate VEGFR-2 phosphorylation, triggering human umbilical vein endothelial cell (HUVEC) proliferation *in vitro* ^53^.

These G _α_-subunit proteins exchange bound GDP for GTP when their associated G protein–coupled receptor (GPCR) is activated ^54, 55^, leading to dissociation of the G-protein heterotrimer—composed of G _α_, G _β_, and G _γ_—from the GPCR and of the activated G _α_ subunit from the G _β_/G _γ_ dimer, ultimately leading to the downstream activation of several cellular pathways.

The mechanism of tumorigenesis associated with these mutations can provide insights into targeted therapeutics. G _α_ is upstream of both the Ras-MAPK and the PI3K-Akt-mTOR, and previous studies of low-flow, lymphatic/venous, vascular malformations implicated the PI3K-Akt-mTOR pathway as the primary driver of these lesions ^56– 59^. Although sirolimus is effective in the treatment of these low-flow lesions ^60– 63^, it has shown little efficacy in vascular tumors, suggesting a distinct pathobiology ^62, 64, 65^. In a study using primary HUVECs expressing mutant *GNA11* and *GNA14*, cells showed no indication of PI3K-Akt-mTOR pathway activation (as measured by pAKT) and instead showed specific activation of the Ras-MAPK pathway (via increased pERK) ^47^, suggesting that a more effective therapy may involve targeting the Ras-MAPK pathway. Recent work suggests that high-flow arteriovenous malformations (AVMs) are also driven by mutations within the Ras-MAPK signaling pathway ^66^. Thus, advances in therapy for vascular tumors may also benefit patients with these high-flow vascular malformations. Somatic mutations in downstream components of this pathway in other vascular tumors further highlight the Ras-MAPK pathway as a primary driver of tumorigenesis in childhood vascular tumors.

## The MAPK pathway

Activation of G _α_ leads to increased RAS activation ^67^. Indeed, a study of sporadic LCHs identified somatic mutations in all three subgroups of the RAS subfamily: *HRAS*, *KRAS*, and *NRAS* ^68^. The activating mutations, which mainly fall at codons 12, 13, and 61, have been established to generate constitutive Ras-MAPK signaling by preventing GTP hydrolysis ^69^. Furthermore, a study of LCHs arising within PWS also identified a p.V600E mutation in *BRAF*, a proto-oncogene directly downstream of Ras in the MAPK pathway chain ^70^. Interestingly, the study also found that both the underlying PWS and the LCH carried mutations in the arginine 183 position of *GNAQ* but that *BRAF* or *RAS* mutation was specific to the LCHs, suggesting that the LCHs arose because of a “second-hit” in *RAS* or *BRAF*.

Mutations in the Fos family of transcription factors, which are among the final components of the Ras-MAPK pathway, have also been identified in childhood vascular tumors. Cytogenetic study of pseudomyogenic hemangioendothelioma (PHE) revealed a t(7;19) (q22;q13) translocation as the sole anomaly in three lesions from one patient ^71^. Later study of this patient and an additional case of PHE revealed that this translocation leads to a *SERPINE1*- *FOSB* fusion gene ^72^. Although vascular endothelial cells demonstrate strong endogenous expression of *SERPINE1*, the *SERPINE1*- *FOSB* disrupts the protein-coding portion of the *SERPINE1* gene, instead generating high levels of *FOSB* mRNA via a promoter switch mechanism ^72– 74^. *FOSB* and *FOS* mutations have also been identified in cases of epithelioid hemangioma (EH). In one study of EH, Antonescu *et al*. identified two fusion genes: *WWTR1*/ *FOSB* and *ZFP36*/ *FOSB* ^75^. It has also been demonstrated that the *FOS* rearrangement leading to the truncation of the FOS protein, specifically to loss of the transactivation domain (TAD), leads to EH in bone.

## Summary

The highlighted genetic discoveries in vascular tumor biology provide novel targets for therapeutics. Indeed, the fact that most of these mutations are present in known cancer-causing pathways means that many medications that are currently approved or under trial for other malignancies may be repurposed for use in vascular tumor therapy.

Mutations in *IDH* are known to cause gliomas, glioblastomas, chondrosarcomas, intrahepatic cholangiocarcinomas, and hematologic malignancies in addition to SCHs ^76^. As such, a number of therapeutics currently under investigation may also be effective in the treatment of SCH. Early results from trials of ivosidenib (AG-120), a novel inhibitor of mutant IDH1, in *IDH1*-mutated acute myelogenous leukemia (AML) indicated an overall response rate of 41.6% and a complete remission rate of 21.6% ^77^. Similarly, results from early trials of enasidenib (AG-221), a novel inhibitor of mutant IDH2, in *IDH2*-mutated AML indicated an overall response rate of 40.3% and a complete remission rate of 19.3% ^78^. These agents are currently under study in a number of clinical trials (ClinicalTrials.gov Identifiers: NCT02074839, NCT02073994, NCT01915498, NCT02577406, NCT02632708, and NCT02677922). Mouse studies have also shown potential for an IDH1 peptide vaccine ^79^, which is currently under trial (ClinicalTrials.gov Identifiers: NCT02454634 and NCT02193347).

Selective inhibition of the Ras-MAPK pathway may provide a novel therapeutic avenue for childhood vascular lesions, which currently have few effective non-surgical options ^80^. The central role of this pathway in tumor pathobiology has necessitated the development of a number of currently available medications, including farnesyl transferase inhibitors such as salirasib, BRAF inhibitors such as vemurafenib, MEK inhibitors such as trametinib, and ERK inhibitors such as ulixertinib, which warrant further study as therapy for childhood vascular tumors. Indeed, Al-Olabi *et al*. demonstrated that treatment of AVMs in *BRAF-*mutant zebrafish with vemurafenib leads to restoration of blood flow in AVMs where it was previously limited ^81^. With promising early results, these therapies hold great potential for the treatment of childhood vascular tumors.
